# Artificial Intelligence in Community-Based Diabetic Retinopathy Telemedicine Screening in Urban China: Cost-effectiveness and Cost-Utility Analyses With Real-world Data

**DOI:** 10.2196/41624

**Published:** 2023-02-23

**Authors:** Senlin Lin, Yingyan Ma, Yi Xu, Lina Lu, Jiangnan He, Jianfeng Zhu, Yajun Peng, Tao Yu, Nathan Congdon, Haidong Zou

**Affiliations:** 1 Department of Eye Disease Prevention and Control, Shanghai Eye Disease Prevention and Treatment Center/Shanghai Eye Hospital Shanghai China; 2 Shanghai Key Laboratory of Ocular Fundus Diseases, Shanghai General Hospital, Shanghai Engineering Center for Visual Science and Photomedicine Shanghai China; 3 Centre for Public Health, Queen's University Belfast Belfast United Kingdom; 4 Orbis International New York, NY United States; 5 Zhongshan Ophthalmic Center, Sun Yat-sen University Guangzhou China

**Keywords:** artificial intelligence, cost, diabetic retinopathy, utility, low- and middle-income countries, screening

## Abstract

**Background:**

Community-based telemedicine screening for diabetic retinopathy (DR) has been highly recommended worldwide. However, evidence from low- and middle-income countries (LMICs) on the choice between artificial intelligence (AI)–based and manual grading–based telemedicine screening is inadequate for policy making.

**Objective:**

The aim of this study was to test whether the AI model is more worthwhile than manual grading in community-based telemedicine screening for DR in the context of labor costs in urban China.

**Methods:**

We conducted cost-effectiveness and cost-utility analyses by using decision-analytic Markov models with 30 one-year cycles from a societal perspective to compare the cost, effectiveness, and utility of 2 scenarios in telemedicine screening for DR: manual grading and an AI model. Sensitivity analyses were performed. Real-world data were obtained mainly from the Shanghai Digital Eye Disease Screening Program. The main outcomes were the incremental cost-effectiveness ratio (ICER) and the incremental cost-utility ratio (ICUR). The ICUR thresholds were set as 1 and 3 times the local gross domestic product per capita.

**Results:**

The total expected costs for a 65-year-old resident were US $3182.50 and US $3265.40, while the total expected years without blindness were 9.80 years and 9.83 years, and the utilities were 6.748 quality-adjusted life years (QALYs) and 6.753 QALYs in the AI model and manual grading, respectively. The ICER for the AI-assisted model was US $2553.39 per year without blindness, and the ICUR was US $15,216.96 per QALY, which indicated that AI-assisted model was not cost-effective. The sensitivity analysis suggested that if there is an increase in compliance with referrals after the adoption of AI by 7.5%, an increase in on-site screening costs in manual grading by 50%, or a decrease in on-site screening costs in the AI model by 50%, then the AI model could be the dominant strategy.

**Conclusions:**

Our study may provide a reference for policy making in planning community-based telemedicine screening for DR in LMICs. Our findings indicate that unless the referral compliance of patients with suspected DR increases, the adoption of the AI model may not improve the value of telemedicine screening compared to that of manual grading in LMICs. The main reason is that in the context of the low labor costs in LMICs, the direct health care costs saved by replacing manual grading with AI are less, and the screening effectiveness (QALYs and years without blindness) decreases. Our study suggests that the magnitude of the value generated by this technology replacement depends primarily on 2 aspects. The first is the extent of direct health care costs reduced by AI, and the second is the change in health care service utilization caused by AI. Therefore, our research can also provide analytical ideas for other health care sectors in their decision to use AI.

## Introduction

Diabetic retinopathy (DR) is a leading cause of blindness worldwide. It often develops 10-15 years after the onset of diabetes and can take several forms—all potentially causing vision loss or blindness, including diabetic macular edema (DME) due to increased retinal vascular permeability and central retinal thickening, retinal ischemia resulting in the damage or death of light-sensing retinal photoreceptors, and proliferative DR, where the growth of fragile new blood vessels causes vitreous hemorrhage and retinal detachment [1].

In 2020, the global burden of DR and sight-threatening DR (STDR) was estimated to be 103 million and 29 million people, respectively, which will increase to 161 million and 45 million by 2045 due to the increasing prevalence of diabetes mellitus (DM) [1,2]. From 1990 to 2010, visual impairment due to DR increased by 64% and blindness by 27% [1], both of which were due to the rising DM prevalence in low- and middle-income countries (LMICs). Several studies have confirmed the benefits of telemedicine screening for DR [3-6]. Compared to no screening and traditional face-to-face screening, telemedicine screening is highly cost-effective in the long term. As a result, telemedicine screening will become the main form of community-based eye disease screening [5]. Some recent studies have suggested that using artificial intelligence (AI) can further reduce the costs of telemedicine screening [3,7-10]. Studies in high-income countries such as the United Kingdom and Singapore have shown that when AI is used in DR screening programs, screening costs can be reduced by up to approximately 20% compared with the costs incurred in manual grading [7-9]. This can be easily understood: from the perspective of a health economic evaluation, the main difference between the AI model and manual grading is that technology costs replace labor costs. Therefore, in settings where labor costs are high, such as in high-income countries, using AI instead of manual grading would save a lot of screening costs, making the screening more cost-effective [10]. However, because labor costs are low in low-income countries, conclusions from high-income countries may not be equally suitable, and evidence from LMICs is inadequate. Therefore, the objective of our community-based telemedicine screening for DR was to examine whether the AI model can be more cost-effective than manual grading in LMICs. We conducted a health economic evaluation by using real-world data from a large community-based telemedicine screening program for DR in Shanghai, China. We expect that this study will provide a reference for policy making with regard to DR screening in the context of low labor costs.

## Methods

### Study Setting

This study was conducted in Shanghai, China, wherein the prevalence of type 2 diabetes among adults was 6.25% between 2016 and 2019 [11]. Since 2010, Shanghai has conducted a teleophthalmology-based DR screening program under which residents can undergo fundus photography at community health service centers. After retinal experts at designated DR diagnosis centers have made a diagnosis based on these images, screening results are fed into the community health service center, where patients are counselled by general practitioners, and medical advice is offered. By 2017, all 250 community health service centers in Shanghai were equipped to participate in this program, and plans had begun to build an AI-assisted DR screening system [12,13]. A convolutional neural network, a type of deep learning model [14], was applied to the problem of diagnosing DR from fundus images, with the aim of replacing retinal experts in DR diagnosis centers with the AI algorithm on a cloud-based server. Since 2020, 56 community health service centers have shifted to an AI-assisted DR screening system. In 2021, these centers screened approximately 40,000 community residents for DR by using AI. To maximize the efficiency of the Shanghai program, community health service centers coordinated voluntary screening for residents of a given community at a particular place and time. Those who were diagnosed with DR at hospitals could still participate in the free annual community screening to monitor disease progression.

### Model Overview

TreeAge Pro (TreeAge Software) was used to build a decision-analytic Markov model to compare the actual cost, effectiveness, and utility of manual grading telemedicine screening and AI-based assessment for DR (Multimedia Appendices 1-4). The incremental cost-effectiveness ratio (ICER) and incremental cost-utility ratio (ICUR) were calculated as the primary results. The effectiveness was defined as years without blindness per 100,000 people with DM, and the utility was evaluated by quality-adjusted life years (QALYs). Although all residents with DM could participate in our community-based screening, the majority were older people [15,16]; therefore, a hypothetical cohort of community residents with DM was followed in the model from the age of 65 years through a total of 30 one-year Markov cycles [5]. The characteristics of the simulated cohort were extracted using the Shanghai Digital Eye Disease Screening Program (Table 1).

**Table 1:** Characteristics of the simulated cohort and comparisons between community health centers using artificial intelligence and those using manual grading.

		All CHCs^a^	CHCs using artificial intelligence	CHCs using manual grading	*P* value
**Community health center characteristics^b^, mean (SD)**					
	Number of full-time or part-time ophthalmologists	1.1 (0.7)	1.1 (0.5)	1.2 (0.7)	.62
	Annual numbers of ophthalmology outpatients	6248.6 (6019.0)	6774.8 (6238.2)	6016.2 (5946.4)	.54
**Screened residents’ characteristics^c^**					
	Age (years), mean (SD)	69.3 (7.2)	69.8 (6.9)	68.8 (7.4)	<.001
	Sex (male), n (%)	15,032 (46.0)	7225 (46.1)	7807 (45.8)	.60
	Duration of diabetes (years), mean (SD)	10.3 (7.7)	9.8 (8.0)	10.7 (7.3)	<.001
	Visual acuity of right eye (logMAR), mean (SD)	0.4 (0.4)	0.4 (0.4)	0.3 (0.4)	<.001
	Visual acuity of left eye (logMAR), mean (SD)	0.4 (0.4)	0.4 (0.4)	0.3 (0.4)	<.001

^a^CHC: community health center.

^b^In 2019, we conducted an investigation of the ophthalmic resources in Shanghai. Out of 250 community health centers, 111 were randomly selected. After matching with the Shanghai Digital Eye Disease Screening program data, 34 community health centers using artificial intelligence and 77 community health centers using manual grading–based telemedicine screening were investigated. We compared both these groups to see whether the community health centers’ characteristics were different between those choosing new technology and those who did not.

^c^A total of 32,695 residents with diabetes were screened in the Shanghai Digital Eye Disease Screening program. Among them, 15,663 residents were in the community health centers with artificial intelligence, and the rest 17,032 were in the community health centers with manual grading. We compared the residents’ characteristics in both groups of community health centers. Although some residents’ characteristics were significantly different owing to the large sample size in the 2 groups, the differences were not clinically meaningful. Therefore, it can be assumed that there is no practical difference between the 2 groups.

Individuals were enrolled as healthy (free from DR) or unhealthy (experiencing DR) and could die due to any reason. According to the English National Screening Program for Diabetic Retinopathy, a Markov model was constructed that included non-STDR, STDR, and DME [5,15-17]. The category was assigned based on the DR grade in the worse eye. During each 1-year cycle, an individual had a risk of progressing to the more severe stage or staying in the same stage. However, the model does not allow returning to an earlier stage even with treatment because of the nature of the disease. Moreover, the treatment can only decrease the probability of progression to the next stage. The prevalence of DR, the incidence of DR (including STDR and DME), transition probabilities, characteristics of DR screening tests, referral and treatment compliance, utility, mortality, and other relevant parameters were collected from published studies specific to Shanghai, other cities in China, and other Asian regions, as well as unpublished data sources (eg, Shanghai Digital Eye Disease Screening Program). The costs of screening, ocular examinations, and treatment were all derived from a real-world eye disease screening program in Shanghai and the unified health care service pricing of the Shanghai Municipal Health Commission. The parameters used in the basic analysis and the ranges used in the sensitivity analyses are listed in detail in Multimedia Appendices 1-4.

### Overview of the Screening Strategies

#### Manual Grading–Based Telemedicine Screening

We invited the entire population with DM living in communities to participate in the DR screening program at local community health centers. All the participants underwent a series of screening tests conducted by trained general practitioners, ophthalmic technicians, optometrists, and ophthalmologists. The screening included a vision acuity test, refraction measurement by an autorefractor, and fundus photography using a non–mydriatic fundus camera. The data were transferred to the corresponding designated diagnosis center through a telemedicine platform after the completion of all the tests. After all the participants in 1 community health center completed the annual screening, the community health center contacted the designated diagnosis center, and 2 retinal experts (ophthalmologists) began to make the diagnosis based on retinal photography. In 2 weeks, screening results were provided as feedback to the community health center, where residents could receive medical advice from the general practitioners. Finally, patients with suspected STDR were referred to specialized ophthalmic hospitals or tertiary hospitals for a detailed re-examination to confirm the diagnosis (Multimedia Appendix 5 shows the screening and referral pathway). Those who were confirmed to have STDR were assumed to receive appropriate treatment and routine clinical care according to the severity of DR.

#### AI-Assisted DR Telemedicine Screening

We invited the entire population with DM living in the community to participate in the DR screening program at the local community health center. The screening process was the same as that described for the manual grading–based telemedicine screening. However, after all the screening tests were completed, the data were transmitted to the AI algorithm on a cloud-based server center through the telemedicine platform. The screening results were provided as feedback immediately. Further management of patients with suspected STDR was the same as that described for manual grading–based telemedicine screening.

### Prevalence and Transition Probabilities

Data on the prevalence and incidence of DR, DME, and STDR were collected from published studies in Shanghai [15,18]. Because Jin et al’s [18] study only reported the 5-year incidence of STDR and DME, the 1-year incidence was calculated based on the formula: r = −log (1 − p)/t, where r represents the 1-year incidence and p represents the cumulative incidence over time interval t [19]. Other transition probabilities were obtained from published studies specific to China, and if few data were available for Chinese patients, data from other Asian regions were used. We searched PubMed and China National Knowledge Infrastructure by using the following combinations of terms: “diabetic retinopathy” AND “progression” OR “transition” AND “Chinese” OR “China.”

### Screening and Intervention Costs

Our study included both direct and indirect costs and analyzed them from a societal perspective. Direct medical costs comprised the charges of screening, examination, and treatment. Direct nonmedical costs consisted of transportation costs related to hospital visits, and indirect costs consisted of family members’ time associated with the visits and their wage loss. All costs were collected in Chinese yuan and then converted into US dollars at an exchange rate of CNY 6.90 per dollar [20]. All cost data are listed in Multimedia Appendices 4 and 6-8.

The screening costs were determined based on the Shanghai Digital Eye Disease Screening Program. The screening costs consisted of the purchase and maintenance costs of equipment, labor costs of medical personnel, transportation fees, and income loss for residents. We calculate the annualized cost for fixed assets by assuming a life span of 5 years and no salvage value. The construction and maintenance costs of the telemedicine platform were based on the Shanghai Digital Eye Disease Screening Program. Based on our field observations, it took 6.2, 3, 3.3, and 4.8 minutes on average for 1 participant to complete registration, visual acuity test autorefraction, and retinal photography, respectively. Theoretically, a team with 4 optometrists could screen approximately 100 participants per day, but under real-world working conditions, this is nearly 30 per day. As the participants in our model were older than 65 years, we assumed that they did not incur wage loss. Moreover, we did not include wage loss for the accompanying family members in the screening costs. Therefore, the total costs per person for manual grading–based and AI-based telemedicine screening were US $10.10 and US $9.60, respectively. Multimedia Appendix 6 shows the detailed composition of the screening costs.

To calculate the costs of the detailed re-examinations after referral, direct medical costs consisted of the costs of ocular examinations and equipment and wages for medical personnel; direct nonmedical costs comprised transportation fees related to the visits; and indirect costs included 1 accompanying family member’s wage loss for time spent and per capita daily income in Shanghai in 2020. The examination costs were the unified pricing of the Shanghai Municipal Health Commission. Because public hospitals are nonprofit institutions, the money from these fees is mainly used to subsidize the cost of health care services. Hence, prices in public hospitals can be used to estimate the direct medical costs. Detailed information on the hospital-based examination costs is provided in Multimedia Appendix 7. It was assumed that the wage loss of the accompanying family member for referral was 0 because the majority of them were older than 65 years.

For treatment costs, direct medical costs included the costs of treatment, equipment, and wages for medical personnel; direct nonmedical costs consisted of costs of transportation related to the visits; and indirect costs included 1 accompanying family member’s wage loss based on time spent and per capita daily income in Shanghai in 2020. In the first year, patients with DME were assumed to have received 3 antivascular endothelial growth factor injections. Photocoagulation or vitrectomy was administered to patients with severe nonproliferative DR or proliferative DR. In the follow-up years, an average of 1 antivascular endothelial growth factor injection was administered, and an annual outpatient review was required for patients with STDR. Direct medical costs were estimated using the prices of health care services in public hospitals. The total economic burden for blind patients in the first year was estimated to be US $8920, which included 53.2% direct medical costs, 6.4% direct nonmedical costs, and 40.4% indirect costs (loss of labor resources for family members and low-vision services costs), and there were only indirect costs in the follow-up years [5,21]. Detailed information on the treatment costs is provided in Multimedia Appendix 8.

### Utility and QALYs

We estimated the utility values for each DR stage (seen in Multimedia Appendix 9) to calculate QALYs. Utility values were based on published studies from China and other Asian countries [21,22]. Because the residents who participated in the screening should have diabetes, utility was assumed to be 0.87 but not 1.0 for people without DR, 0.79 for those with non-STDR, and 0.7 for those with STDR (including severe nonproliferative DR, proliferative DR, and DME). The utility value for people with blindness was assumed to be 0.55 [22]. All the values for the base case and sensitivity analyses are listed in Multimedia Appendix 3.

### Compliance

Compliance with referral to specialized ophthalmic hospitals or tertiary hospitals for a full examination among patients screened for signs of STDR was assumed to be 50.4% for manual grading–based telemedicine screening, according to our investigation in Shanghai [23]. However, compliance with AI-based telemedicine screening was unclear. Because only 1 published study suggested that adopting an AI-assisted diagnosis model in DR screening may impact the participants’ adherence to ophthalmic care [24], the evidence is insufficient. Therefore, we assumed that compliance with referral in AI-based telemedicine screening was the same as that in manual grading–based telemedicine screening, while we set a wide range (±25%) for sensitivity analysis (Multimedia Appendix 3).

### Screening Accuracy

The accuracy of AI-based telemedicine screening was extracted from published studies specific to the AI-assisted screening model conducted in Shanghai based on the current dominant architecture of convolutional neural networks (Multimedia Appendix 10) [25]. Briefly, the sensitivity was 80.47% (95% CI 75.07%-85.14%) and the specificity was 97.96% (95% CI 96.75%-98.81%) for STDR [25]. In our screening program, 2 experienced ophthalmologists were employed to make the diagnoses based on the retinal images. Furthermore, the accuracy of the manual grading–based telemedicine screening was assumed to be 100%, which was in accordance with the DR diagnosis criteria [8,9,26,27]. However, as described in some other studies, since trained graders instead of ophthalmologists performed the grading and diagnosis [5,7] in the sensitivity analysis, we adopted the accuracy range of the manual grading based on the Singaporean study (Multimedia Appendix 3) [7].

### Other Parameters

The natural age-specific mortality rates of the general Chinese population reported by Zhang and Wei [28] were used in this study. Increased odds of mortality were assumed for people without DR but with DM, non-STDR, STDR, and blindness (Multimedia Appendix 3) [29,30]. Both costs and health state utilities were discounted at a 3.5% annual rate in the base analysis, following the National Institute for Health and Care Excellence recommendations [31]. For the cost-effectiveness threshold, 2 thresholds representing cost-effectiveness and high cost-effectiveness were used according to the World Health Organization recommendations [5,21]. Among the interventions improving the patients’ utilities, those that cost less than the gross domestic product (GDP) per capita are defined as highly cost-effective, those that cost 1-3 times the GDP per capita are defined as cost-effective, and those that cost more than 3 times the GDP per capita are determined as not cost-effective [32]. On the contrary, among the interventions reducing the participants’ utilities, among those saving costs higher than 3 times, the GDP per capita was defined as highly cost-effective; among those saving between 1 and 3 times, the GDP per capita was defined as cost-effective; and those costing less than the GDP per capita were determined as not cost-effective [32]. As the GDP per capita in Shanghai in 2020 was reported to be US $22,600, the thresholds in this study were defined as US $22,600 and US $67,800 [33].

### Outcomes

The ICER and ICUR were calculated as the difference in the total costs between the AI-assisted and manual grading telemedicine screening divided by the difference in the total years without blindness and the QALYs between the 2 conditions, respectively. Values for the AI-assisted screening cohort minus those for the manual grading screening cohort, which were set as the baseline, were calculated as the differences.

### Sensitivity Analysis

Extensive 1-way deterministic and probabilistic sensitivity analyses were performed to calculate the uncertainties of the base-case results. A variation of 10% was adopted because probability-related statistics (ie, utility, prevalence, sensitivity, specificity, transition probability, and compliance) were mainly derived from previously published studies. For the influence of AI use on compliance with referral, a range of 25% was used. A large floating range of 50% was adopted for these costs. In addition, we adopted the accuracy range for manual grading according to the Singaporean study (Multimedia Appendix 3) to account for the influence of trained graders performing the grading and diagnosis instead of retinal experts [7]. A probabilistic sensitivity analysis was conducted using Monte Carlo simulation for 10,000 simulations to assess the robustness of the base case analysis. Beta distributions were adopted for probability-related data and utility values, gamma distributions were used for costs, and log-normal distributions were used for odds ratios. The methods and results conformed to the Consolidated Health Economic Evaluation Reporting Standards (2022) (Multimedia Appendix 11).

### Ethics Approval

This study was mainly based on the secondary analyses of published data. Written informed consent was obtained from all the participants. All the study data were anonymous. There were no compensation fees for the participants. This study was approved by the Institutional Review Board of the Shanghai General Hospital (2022SQ272) and Shanghai Eye Diseases Prevention and Treatment Center (2022SQ007).

## Results

The cost-effectiveness and cost-utility analyses showed that AI-based telemedicine screening was dominated by manual grading–based telemedicine screening in Shanghai (Table 2). In the manual grading–based telemedicine screening, a community resident with DM would incur a total cost of US $3265.40, including screening, hospital referral for confirmation, and treatment as needed, with 9.83 years without blindness and 6.753 QALYs. In the AI-based telemedicine screening, a community resident with DM would incur a total cost of US $3182.50, with 9.80 years without blindness and 6.748 QALYs. Therefore, compared with the cost of manual grading–based telemedicine screening, that of the AI-based telemedicine screening model was 2.5% lower, while the years without blindness was 0.3% less, and the QALYs were 0.1% less.

**Table 2:** Base-case cost-effectiveness and cost-utility results^a^.

	Costs per person (USD)	Incremental costs per 100,000 people screened (USD)	Years without blindness per person	Incremental years without blindness per 100,000 people screened	QALY^b^ per person	Incremental quality-adjusted life years per 100,000 people screened	ICER^c^ (USD)	ICUR^d^ (USD)
AI^e^-assisted model	3182.47	–8,289,840.65	9.80	–3121.32	6.748	–544.78	2553.39	15,216.96
Manual grading	3265.37	N/A^f^	9.83	N/A	6.753	N/A	N/A	N/A

^a^Costs, years without blindness, and quality-adjusted life years are lifetime values per person, whereas incremental costs, incremental years without blindness, incremental cost-effectiveness ratio, incremental quality-adjusted life years, and incremental cost-utility ratio are calculated against the manual grading–based telemedicine screening scenario per 100,000 people screened.

^b^QALY: quality-adjusted life year.

^c^ICER: incremental cost-effectiveness ratio.

^d^ICUR: incremental cost-utility ratio.

^e^AI: artificial intelligence.

^f^N/A: not applicable.

**Figure 1:**
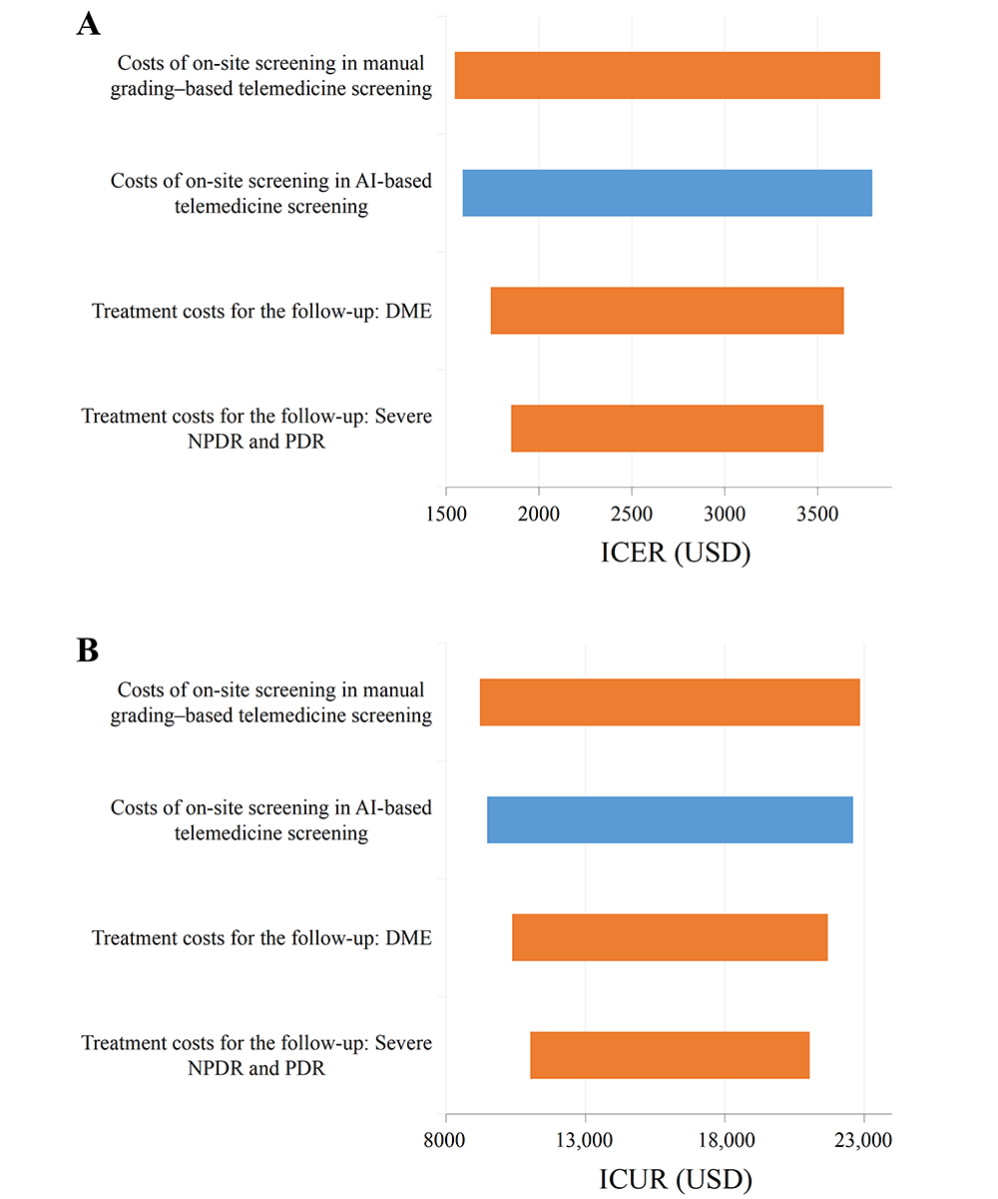
One-way deterministic sensitivity analysis (Tornado diagram). A. One-way sensitivity analysis of cost-effectiveness. B. One-way sensitivity analysis of cost-utility. Since negative values of incremental cost-effectiveness ratio or incremental cost-utility ratio might occur due to the change of compliance with referral after the adoption of artificial intelligence (multiplier), detailed results have been shown in Multimedia Appendix 12 separately. Therefore, in this Tornado diagram, the impact of the change of compliance with referral after the adoption of artificial intelligence (multiplier) is not shown. AI: artificial intelligence; DME: diabetic macular edema; ICER: incremental cost-effectiveness ratio; ICUR: incremental cost-utility ratio; NPDR: nonproliferative diabetic retinopathy; PDR: proliferative diabetic retinopathy.

Our results showed that by replacing manual grading–based telemedicine screening with AI-based telemedicine screening, 1 participant could save US $15,216.96 but needed to lose 1 more QALY (ICUR=US $15,216.96), indicating that AI-based telemedicine screening was not cost-effective as in Shanghai in 2020; at least US $22,600 (GDP per capita) should be saved if 1 more QALY is lost due to the shift in interventions. A 1-way deterministic sensitivity analysis of cost-effectiveness and cost-utility analyses indicated that the impact of the adoption of AI on compliance with referral, costs of on-site screening in manual grading–based telemedicine screening, costs of on-site screening in AI-based telemedicine screening, treatment costs for the follow-up of patients with DME, and treatment costs for the follow-up of patients with severe nonproliferative DR and proliferative DR were the 5 most influential variables. In particular, according to the cost-utility analysis, if the adoption of AI could improve compliance with referrals by 7.5%, the AI-assisted model might be cost-effective; if compliance was improved by 17.5%, the AI-assisted model might be highly cost-effective; and if compliance was improved by 25%, the AI-assisted model might be the absolutely dominant strategy, as it could save costs and increase the years without blindness and QALYs (Multimedia Appendix 12). Moreover, the increase in the costs of on-site screening in manual grading–based telemedicine screening and the decrease in the costs of on-site screening in AI-based telemedicine screening might help the AI-based telemedicine screening to be cost-effective (Figure 1). The detailed sensitivity analysis results of the other parameters are shown in Multimedia Appendices 13 and 14.

**Figure 2:**
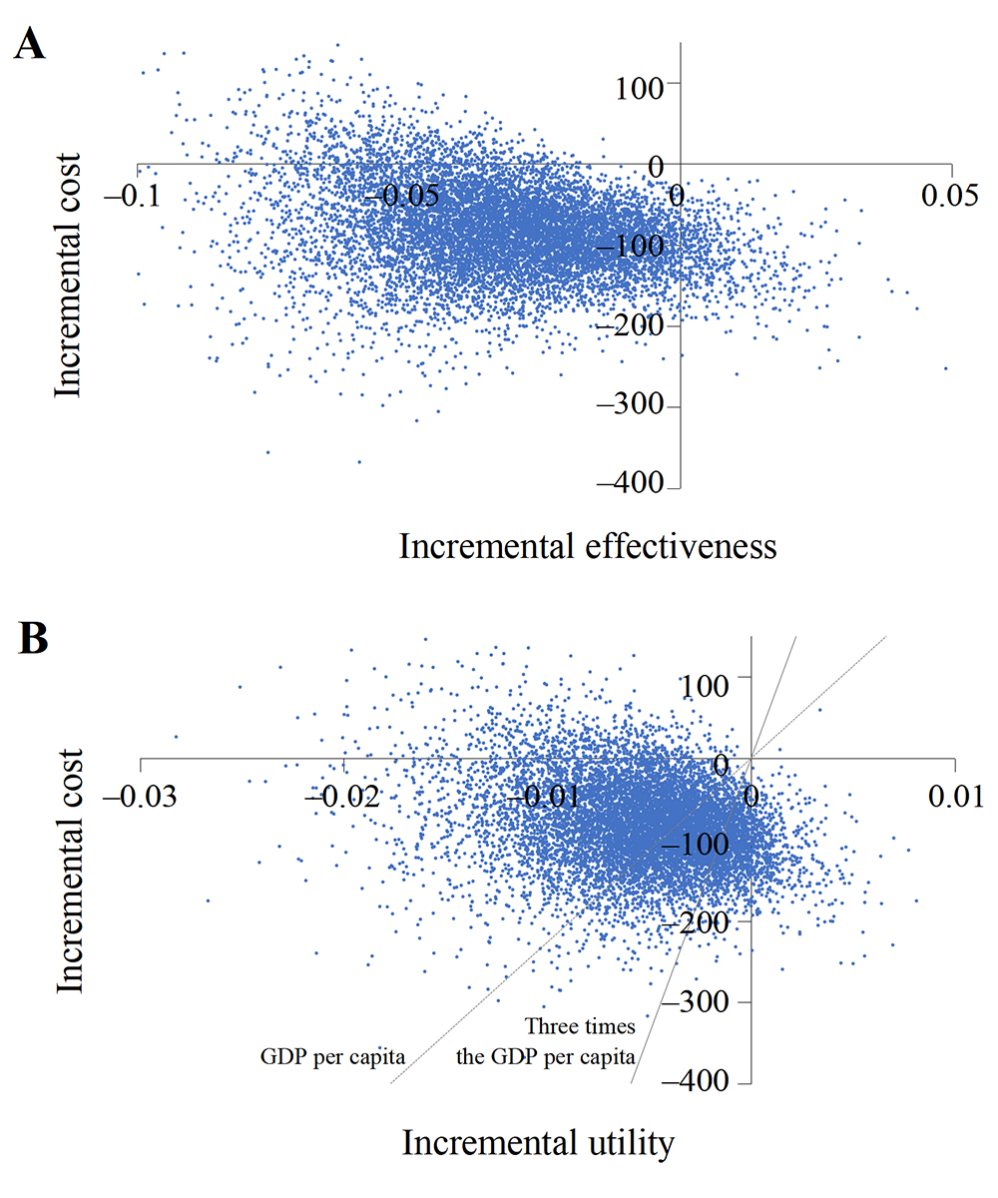
Probabilistic sensitivity analysis. A. Probabilistic sensitivity analysis of cost-effectiveness. B. Probabilistic sensitivity analysis of cost-utility. GDP: gross domestic product.

**Figure 3:**
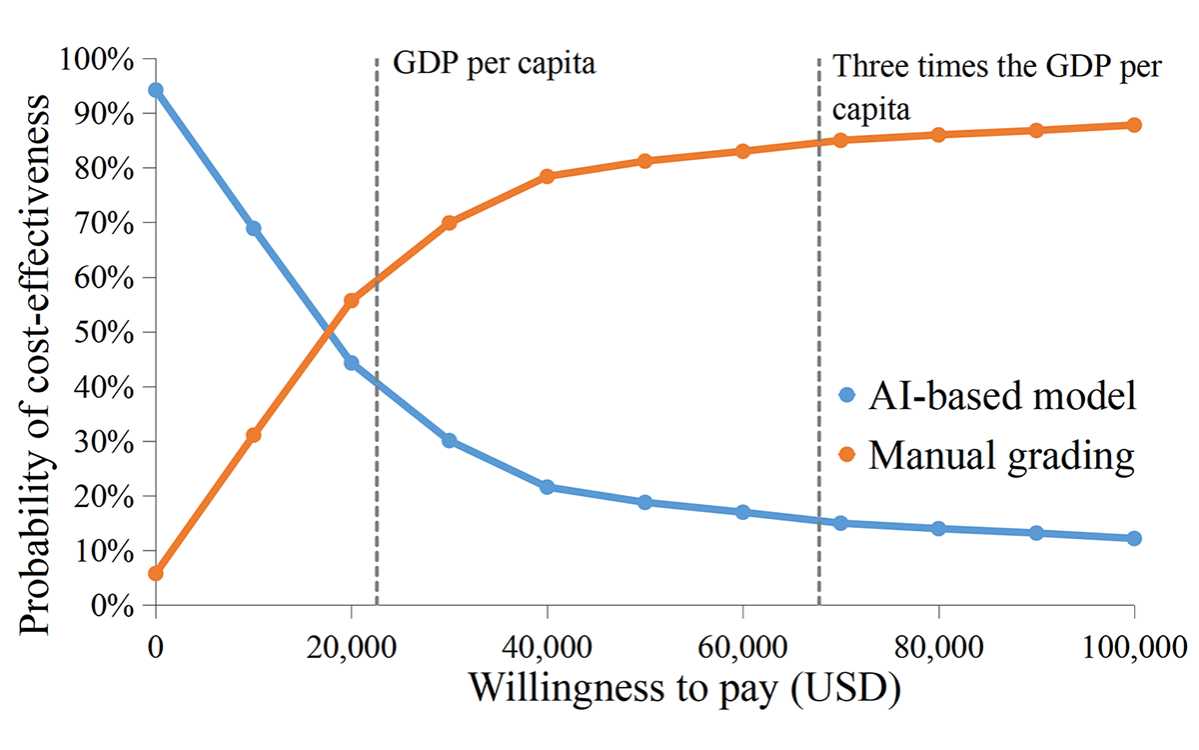
Cost-effectiveness acceptability curves. AI: artificial intelligence; GDP: gross domestic product.

Probabilistic sensitivity analysis showed that the base-case ICER and ICUR were robust to randomly distributed parameters (Figure 2). We obtained cost-effectiveness acceptability curves by taking 10,000 random draws (Figure 3). This means that when both AI-based and manual grading–based telemedicine screening were available, manual grading–based telemedicine screening was the dominant strategy in 60.6% of the simulations under the threshold of GDP per capita (US $22,600) and in 84.5% of the simulations under the threshold of 3 times the GDP per capita (US $67,800).

## Discussion

### Principal Findings

This study presents one of the first health economic evaluations in the context of low labor costs in an LMIC setting of competing telemedicine models for community-based DR screening using manual grading and AI models. In line with previous studies [7,8], our analysis was based on an established telemedicine screening program. We showed that, in this context, the value of AI-based telemedicine DR screening depended heavily on the referral compliance of patients with suspected STDR. If this compliance did not increase, AI-based telemedicine DR screening would not be more cost-effective than manual grading–based telemedicine DR screening because it would decrease the long-term screening effectiveness and individuals’ health utility and not save enough costs.

Prior studies in Singapore and the United Kingdom [7,8] showed that replacing manual grading with an AI model for DR screening led to a 12%-20% cost reduction. A study in Scotland reported an even greater cost reduction of 46.7% [9]. These studies were conducted in high-income countries in the context of high labor costs. However, China’s national conditions differ from those in high-income countries. One of the most important differences is that the labor costs of the medical staff are much lower in China. For example, in Singapore, the labor cost for DR grading is US $26 per participant [7], which is over 20 times that of the labor cost for manual grading in Shanghai. Consequently, in the context of low labor costs, a reduction in screening costs resulting from the use of AI solutions is limited in China.

In the Shanghai program, the on-site screening cost for 1 participant, including screening examinations and diagnosis, was US $10.10 in the manual grading model and US $9.60 in the AI-assisted model—a reduction of only 5%. Moreover, the labor costs of the medical staff in our screening program were among the highest in China. For example, according to the Wenzhou ophthalmologic screening program, the labor cost for on-site screening examinations and diagnosis of glaucoma was US $1.70 per participant, which was about a quarter of the labor costs in our Shanghai program (US $7.20 per participant for manual grading–based telemedicine screening, Multimedia Appendix 6) [21], and according to the Finance Department and Procurement Center of Beijing Tongren Hospital, Beijing Tongren Eye Centre Ocular Reading Centre, and China Intelligent Ophthalmology Big Data Research Center, the labor costs for on-site screening examinations and diagnosis were US $1.75 per participant for traditional face-to-face screening and US $0.80 per participant for manual grading–based telemedicine screening [5], which is only one-ninth of the labor costs in our Shanghai program (US $7.20 per participant for manual grading–based telemedicine screening, Multimedia Appendix 6). Because the main difference between the cost components of the AI-assisted model and those of the manual grading model is that equipment and telemedicine platform costs replace labor costs, in settings where labor costs are extremely low, cost reduction via the adoption of AI is expected to be even lesser. Therefore, AI-assisted DR screening is less cost-effective in other urban areas of China.

Our sensitivity analysis confirmed these results. The on-site screening costs of both manual grading and AI-assisted models are among the most influential variables. An increase in the on-site screening costs of manual grading by 50% or a decrease in the on-site screening costs of the AI-assisted model by 50% may help AI-based telemedicine screening be cost-effective. In other words, the gap between the on-site screening costs of the manual grading model and the AI-assisted model is the key point. Moreover, the cost of AI software was only 7% of the on-site costs in AI-based telemedicine screening (Multimedia Appendix 6). Therefore, even if the AI software were completely free, a 50% reduction in the on-site screening costs of the AI-assisted model would not be achieved. As a result, unless the labor costs of medical staff increase dramatically in the future, the AI-assisted model will be hardly cost-effective in Shanghai, holding all the other conditions constant.

However, there was one exception to this. Our sensitivity analysis shows that if the referral compliance of patients with suspected STDR increased after the adoption of AI even to a small extent, then the AI-assisted model would be cost-effective. A study in Missouri [24] suggested that after the adoption of the automated retinal image assessment system, which is based on AI, the rates of completed referral eye examinations at 3, 6, and 12 months after screening increased from 9.4% to 32.6%, from 13.4% to 46.7%, and from 18.7% to 55.4%, respectively [24]. However, relevant evidence is still inadequate; therefore, it is difficult to determine whether this improvement is an isolated case. Previously, we implemented a discrete choice experiment in Shanghai to measure individuals’ preferences for AI-based screening [34]. The results suggested that the impact of the adoption of AI on individuals’ preferences may be bidirectional. On the one hand, algorithm aversion should be noted, which means that compared to manual grading, the residents were in disfavor of the AI-assisted screening technology [34,35]. On the other hand, the immediate feedback of retinal screening results by the adoption of AI could increase the individuals’ preferences and have profound effects on participants’ follow-up behavior [24,34]. Nevertheless, there is still a lack of empirical studies on the association between the results of feedback efficiency and residents’ referral compliance.

This study has several strengths. This study may provide a reference for policy making in planning community-based DR screening in LMICs by modelling 2 practical telemedicine screening models for DR by using real-world data from an ongoing program in urban China. In addition, we conducted a sensitivity analysis of our models within wide ranges and identified the most influential variables affecting the decision to use AI and manual grading in telemedicine screening. Therefore, our conclusions provide practical value in the policy-making process regarding when to deploy AI-assisted diagnostic technology.

### Limitations

Our study had several limitations despite its numerous strengths. Most notably, we only compared the models for centralized screening. Other models must also be considered going forward. For example, in Shanghai, some community health service centers are beginning to provide DR screening as part of their outpatient services for patients with diabetes. This change in the model may impact both the costs and patients’ compliance, thus altering the results of health economic evaluations such as ours. Second, our comparison is based on the premise that both human- and AI-based models are available and affordable. However, in some remote regions, due to the lack of human resources, manual screening for eye disease may be impractical, and AI-based screening, if available, may be the only option. Third, our study is mainly based on empirical data from Shanghai; therefore, it cannot be representative of the whole of China because of the huge regional and medical care differences between urban and rural areas. Therefore, there is an urgent need for more extensive and in-depth studies. However, as we have discussed above, the labor costs of medical staff in Shanghai are among the highest in China, and AI-based telemedicine screening will become even more less cost-effective if the labor cost of medical staff is further reduced. Therefore, our findings can be extrapolated within the Chinese context.

### Conclusion

Our study may provide a reference for policy making in planning community-based telemedicine screening for DR in LMICs. Our findings indicate that unless the referral compliance of patients with suspected STDR increases, the adoption of the AI model may not further improve the value of telemedicine screening compared to that of manual grading in LMICs. The main reason is that in the context of low labor costs, the direct health care costs saved by replacing manual grading with AI are limited, and screening effectiveness will decrease. In conclusion, our study suggests that the magnitude of the value generated by this technology replacement depends mainly on 2 aspects. The first is the extent of direct health care costs reduced by using AI, and the second is the change in health care service utilization caused by using AI. Therefore, our research also provides analytical ideas for other health care sectors in addition to eye care when deciding whether to use AI.

## Appendix

Multimedia Appendix 1 Prevalence and uncertainty ranges of different stages of diabetic retinopathy.

Multimedia Appendix 2 Variation range and distributions assumed for the transitional probabilities before and after treatment for the different diabetic retinopathy stages.

Multimedia Appendix 3 Variation range and distributions assumed for compliance, utilization, mortality, and other parameters.

Multimedia Appendix 4 Variation range and distributions assumed for screening cost and medical cost of treating diabetic retinopathy at different stages.

Multimedia Appendix 5 Care pathways for both artificial intelligence–based and manual grading–based telemedicine screening.

Multimedia Appendix 6 Cost composition of manual grading–based and artificial intelligence–based telemedicine screening.

Multimedia Appendix 7 Cost composition of full examination.

Multimedia Appendix 8 Cost composition of treatment for patients with sight-threatening diabetic retinopathy.

Multimedia Appendix 9 Markov model for diabetic retinopathy.

Multimedia Appendix 10 Accuracy of the artificial intelligence–assisted model used in Shanghai.

Multimedia Appendix 11 The Consolidated Health Economic Evaluation Reporting Standards 2022 checklist.

Multimedia Appendix 12 Deterministic sensitivity analysis results of change of compliance with referral after the adoption of artificial intelligence (multiplier).

Multimedia Appendix 13 Sensitivity analysis results for incremental cost-effectiveness ratio.

Multimedia Appendix 14 Sensitivity analysis results for incremental cost-utility ratio.

## Abbreviations

AI: artificial intelligence
DM: diabetes mellitus
DME: diabetic macular edema
DR: diabetic retinopathy
GDP: gross domestic product
ICER: incremental cost-effectiveness ratio
ICUR: incremental cost-utility ratio
LMICs: low- and middle-income countries
QALY: quality-adjusted life year
STDR: sight-threatening diabetic retinopathy
