# Supplementary material for: Artificial Intelligence in Community-Based Diabetic Retinopathy Telemedicine Screening in Urban China: Cost-effectiveness and Cost-Utility Analyses With Real-world Data
Source: JMIR Public Health Surveill. 2023 Feb 23;9:e41624. doi: 10.2196/41624 (PMC9999255; doi:10.2196/41624)
Supplement: Multimedia Appendix 1 [file publichealth_v9i1e41624_app1.docx]

**Appendix 1. Prevalence and uncertainty ranges of DR health states**

|  | | **Prevalence** | **Source** | **Range for one-way sensitivity analysis** | **Distributions used in the probabilistic sensitivity analysis** |
| --- | --- | --- | --- | --- | --- |
| **Non-STDR** |  | 14.71% | Calcluated from [1] | ±10% (13.24%, 16.18%) | Beta(590, 3421) |
| **STDR** | **Severe NPDR and PDR** | 0.85% | Calcluated from [1] | ±10% (0.76%,0.93%) | Beta(34, 3977) |
|  | **DME** | 3.37% | Calcluated from [1] | ±10% (3.03%, 3.70%) | Beta(135, 3876) |
| **Blindness** |  | 0.27% | Unpublished data from Shanghai in 2021 | ±10% (0.24%, 0.29%) | Beta(11, 4124) |

DR= diabetic retinopathy. STDR= sight-threatening DR. NPDR= nonproliferative diabetic retinopathy. PDR= proliferative diabetic retinopathy.

DME= diabetic macular edema.

1. He J, Xu X, Zhu J, Zhu B, Zhang B, Lu L, He X, Bai X, Xu X, Zou H. Lens Power, Axial Length-to-Corneal Radius Ratio, and Association with Diabetic Retinopathy in the Adult Population with Type 2 Diabetes. Ophthalmology 2017 Mar; 124(3):326-335.
