# Supplementary material for: Artificial Intelligence in Community-Based Diabetic Retinopathy Telemedicine Screening in Urban China: Cost-effectiveness and Cost-Utility Analyses With Real-world Data
Source: JMIR Public Health Surveill. 2023 Feb 23;9:e41624. doi: 10.2196/41624 (PMC9999255; doi:10.2196/41624)
Supplement: Multimedia Appendix 2 [file publichealth_v9i1e41624_app2.docx]

**Appendix 2. Variation range and distributions assumed for the transitional probabilities before and after treatment for DR stages**

|  | **Untreated** | | | | **After treatment** | | | |
| --- | --- | --- | --- | --- | --- | --- | --- | --- |
|  | Prob. | Source | Range for sensitivity analysis | Distributions used in the probabilistic sensitivity analysis | Prob. | Source | Range for sensitivity analysis | Distributions used in the probabilistic sensitivity analysis |
| Normal to non-STDR | 11.89% | Calculated from [1] | ±10% (10.70%, 13.08%) | Beta(38.3, 283.7) | NA |  |  |  |
| Non-STDR to Severe NPDR and PDR | 9.00% | [2] | ±10% (8.10%, 9.90%) | Beta(255.25, 2614.75) | NA |  |  |  |
| Non-STDR to DME | 9.23% | Calculated from [1] | ±10% (8.31%, 10.16%) | Beta(29.8, 293.2) | NA |  |  |  |
| DME to blindness | 5.00% | [3] | ±10% (4.5%, 5.5%) | Beta(94.95,1804.05) | 3.00% | [3] | ±10% (2.7%, 3.3%) | Beta(96.97,3135.36) |
| Severe NPDR and PDR to blindness | 9.00% | [3] | ±10% (8.1%, 9.9%) | Beta(90.91,919.2) | 2.00% | [3] | ±10% (1.8%, 2.2%) | Beta(97.98,4801.02) |

DR= diabetic retinopathy. STDR= sight-threatening DR. NPDR= nonproliferative diabetic retinopathy. PDR= proliferative diabetic retinopathy.

DME= diabetic macular edema.

1. Jin P, Peng J, Zou H, wang W, Fu J, Bai X, Xu X, Zhang X. A 5-year prospective study of type 2 diabetes patients in Shanghai Xinjing Community 1.The incidence and risk factors of diabetic retinopathy and diabetic macular edema in Chinese type 2 diabetes residents. Chin J Exp Ophthalmol. (in Chinese) 2016; 34(4):363-367
2. Nguyen HV, Tan GSW, Tapp RJ, Mital S, Ting DSW, Wong HT, Tan CS, Laude A, Tai ES, Tan NC, Finkelstein EA, Wong TY, Lamoureux EL. Cost-effectiveness of a National Telemedicine Diabetic Retinopathy Screening Program in Singapore. Ophthalmology 2016 Dec; 123(12):2571-2580
3. Li R, Yang Z, Zhang Y, Bai W, Du Y, Sun R, Tang J, Wang N, Liu H. Cost-effectiveness and cost-utility of traditional and telemedicine combined population-based age-related macular degeneration and diabetic retinopathy screening in rural and urban China. Lancet Reg Health West Pac 2022 Jun; 23:100435
