# Supplementary material for: Artificial Intelligence in Community-Based Diabetic Retinopathy Telemedicine Screening in Urban China: Cost-effectiveness and Cost-Utility Analyses With Real-world Data
Source: JMIR Public Health Surveill. 2023 Feb 23;9:e41624. doi: 10.2196/41624 (PMC9999255; doi:10.2196/41624)
Supplement: Multimedia Appendix 3 [file publichealth_v9i1e41624_app3.docx]

**Appendix 3. Variation range and distributions assumed for compliance, utilization, mortality, and other parameters**

|  | **Base-case values** | **Source** | **Ranges for sensitivity analysis** | **Probability distribution for sensitivity analysis** |
| --- | --- | --- | --- | --- |
| Compliance with |  |  |  |  |
| *Referral* | 54% | [1] | ±10% (48.6%, 59.4%) | Beta(1812, 1550) |
| *Treatment* | 60% | [2] | ±10% (54%, 66%) | Beta(39.4, 26.27) |
| Change of compliance with referral after the adoption of AI (Multiplier) | 1.0 | Assumed | ±25% (0.75, 1.25) | Lognomal(0, 0.13) |
| Utility |  |  |  |  |
| *No DR* | 0.87 | [3] | ±10% (0.783, 0.957) | Beta(12.13, 1.81) |
| *Non-STDR* | 0.79 | [3] | ±10% (0.711, 0.869) | Beta(20.21, 5.37) |
| *Severe NPDR and PDR* | 0.7 | [3] | ±10% (0.63, 0.77) | Beta(29.3, 12.56) |
| *DME* | 0.7 | [3] | ±10% (0.63, 0.77) | Beta(29.3, 12.56) |
| *Blindness* | 0.55 | [3], [4] | ±10% (0.495, 0.605) | Beta(44.45, 36.37) |
| Discount rate for costs and benefits (base-case analysis) | 3.50% |  | None defined |  |
| Accuracy of AI-assisted model |  |  |  |  |
| *No DR called STDR* | 0% | Calculated from [5] | 0, 1% | Beta(0, 411) |
| *Non-STDR called STDR* | 4.03% | Calculated from [5] | ±10% (3.63%, 4.43%) | Beta(17, 405) |
| *STDR called STDR* | 80.47% | Calculated from [5] | ±10% (72.42%, 88.52%) | Beta(206, 50) |
| Accuracy of manual grading |  |  |  |  |
| *No DR called STDR* | 0% | Assumed | 0, 1% | None defined |
| *Non-STDR called STDR* | 0% | Assumed | 0, 1% | None defined |
| *STDR called STDR* | 100.00% | Assumed | 90%, 100% | None defined |
| Mortality rates for age groups |  |  |  |  |
| *50–54 years* | 0.36% | [6] | None defined | None defined |
| *55–59 years* | 0.52% | [6] | None defined | None defined |
| *60–64 years* | 0.85% | [6] | None defined | None defined |
| *65–69 years* | 1.42% | [6] | None defined | None defined |
| *70–74 years* | 3.15% | [6] | None defined | None defined |
| *75–79 years* | 4.86% | [6] | None defined | None defined |
| *80–84 years* | 8.93% | [6] | None defined | None defined |
| Increased mortality risk (Multiplier) |  |  |  |  |
| *DM* | 1.8 | [7], [8] | None defined | None defined |
| *Non-STDR* | 1.49 | [7] | None defined | None defined |
| *STDR* | 1.76 | [7] | None defined | None defined |
| *DME* | 1.76 | [7] | None defined | None defined |
| *Blindness* | 2.34 | [7] | None defined | None defined |

AI=artificial intelligence. DR= diabetic retinopathy. STDR= sight-threatening DR. NPDR= nonproliferative diabetic retinopathy. PDR= proliferative diabetic retinopathy. DME= diabetic macular edema.

1. Zhu X, Xu Y, Lu L, Zou H. Patients' perspectives on the barriers to referral after telescreening for diabetic retinopathy in communities. BMJ Open Diabetes Res Care 2020 Mar; 8(1):e000970
2. Li R, Yang Z, Zhang Y, Bai W, Du Y, Sun R, Tang J, Wang N, Liu H. Cost-effectiveness and cost-utility of traditional and telemedicine combined population-based age-related macular degeneration and diabetic retinopathy screening in rural and urban China. Lancet Reg Health West Pac 2022 Jun; 23:100435
3. Nguyen HV, Tan GSW, Tapp RJ, Mital S, Ting DSW, Wong HT, Tan CS, Laude A, Tai ES, Tan NC, Finkelstein EA, Wong TY, Lamoureux EL. Cost-effectiveness of a National Telemedicine Diabetic Retinopathy Screening Program in Singapore. Ophthalmology 2016 Dec; 123(12):2571-2580
4. Tang J, Liang Y, O'Neill C, Kee F, Jiang J, Congdon N. Cost-effectiveness and cost-utility of population-based glaucoma screening in China: a decision-analytic Markov model. The Lancet Global Health 2019 Jul; 7(7):e968-e978
5. Zhang W, Li D, Wei Q, Ding D, Meng L, Wang Y, Zhao X, Chen Y. The Validation of Deep Learning-Based Grading Model for Diabetic Retinopathy. Front Med (Lausanne) 2022; 9:839088
6. Zhang W, Wei M. The Evaluation of the Mortality and Life Expectancy of Chinese Population. Population Journal. (in Chinese) 2016; 38(3):18-28
7. Vijan S, Hofer T P, Hayward R A. Cost-utility analysis of screening intervals for diabetic retinopathy in patients with type 2 diabetes mellitus. JAMA 2000 Feb 16; 283(7):889-96
8. Bragg F, Holmes MV, Iona A, Guo Y, Du H, Chen Y, Bian Z, Yang L, Herrington W, Bennett D, Turnbull I, Liu Y, Feng S, Chen J, Clarke R, Collins R, Peto R, Li L, Chen Z, China Kadoorie Biobank Collaborative Group. Association Between Diabetes and Cause-Specific Mortality in Rural and Urban Areas of China. JAMA 2017 Jan 17; 317(3):280-289
