# Supplementary material for: Artificial Intelligence in Community-Based Diabetic Retinopathy Telemedicine Screening in Urban China: Cost-effectiveness and Cost-Utility Analyses With Real-world Data
Source: JMIR Public Health Surveill. 2023 Feb 23;9:e41624. doi: 10.2196/41624 (PMC9999255; doi:10.2196/41624)
Supplement: Multimedia Appendix 4 [file publichealth_v9i1e41624_app4.docx]

**Appendix 4. Variation range and distributions assumed for screening cost and medical cost of treating DR at different stages**

|  | Treatment costs for the first year ($/person) | Range for sensitivity analysis and assigned distribution ($/person) | Probability distribution for sensitivity analysis | Annual medication costs during follow-up years ($/person) | Range for sensitivity analysis ($/person) | Probability distribution for sensitivity analysis |
| --- | --- | --- | --- | --- | --- | --- |
| Screening |  |  |  |  |  |  |
| *Telemedicine screening* | 10.1 | ±50% (5.05, 15.15) | Gamma(16, 1.58) | NA |  |  |
| *AI-assisted screening* | 9.6 | ±50% (4.8, 14.4) | Gamma(16, 1.67) | NA |  |  |
| Full ophthalmologic examination | 57 | ±50% (28.5, 85.5) | Gamma(16, 0.28) | NA |  |  |
| Treatment costs |  |  |  |  |  |  |
| *Severe NPDR and PDR* | 926.3 | ±50% (463.2, 1389.5) | Gamma(16, 0.02) | 960.9 | ±50% (480.45, 1441.35) | Gamma(16, 0.02) |
| *DME* | 2782.2 | ±50% (1391.1, 4173.3) | Gamma(16, 0.01) | 960.9 | ±50% (480.45, 1441.35) | Gamma(16, 0.17) |
| *Blindness* | 8920 | ±50% (4460, 13380) | Gamma(16, 0.02) | 3600 | ±50% (1800, 5400) | Gamma(16, 0.004) |

DR= diabetic retinopathy. STDR= sight-threatening DR. NPDR= nonproliferative diabetic retinopathy. PDR= proliferative diabetic retinopathy. DME= diabetic macular edema.
