# Supplementary figures and images for: Artificial Intelligence in Community-Based Diabetic Retinopathy Telemedicine Screening in Urban China: Cost-effectiveness and Cost-Utility Analyses With Real-world Data
Source: JMIR Public Health Surveill. 2023 Feb 23;9:e41624. doi: 10.2196/41624 (PMC9999255; doi:10.2196/41624)

**Appendix 5. Care pathways for both AI-based and manual grading-based telemedicine screening.**


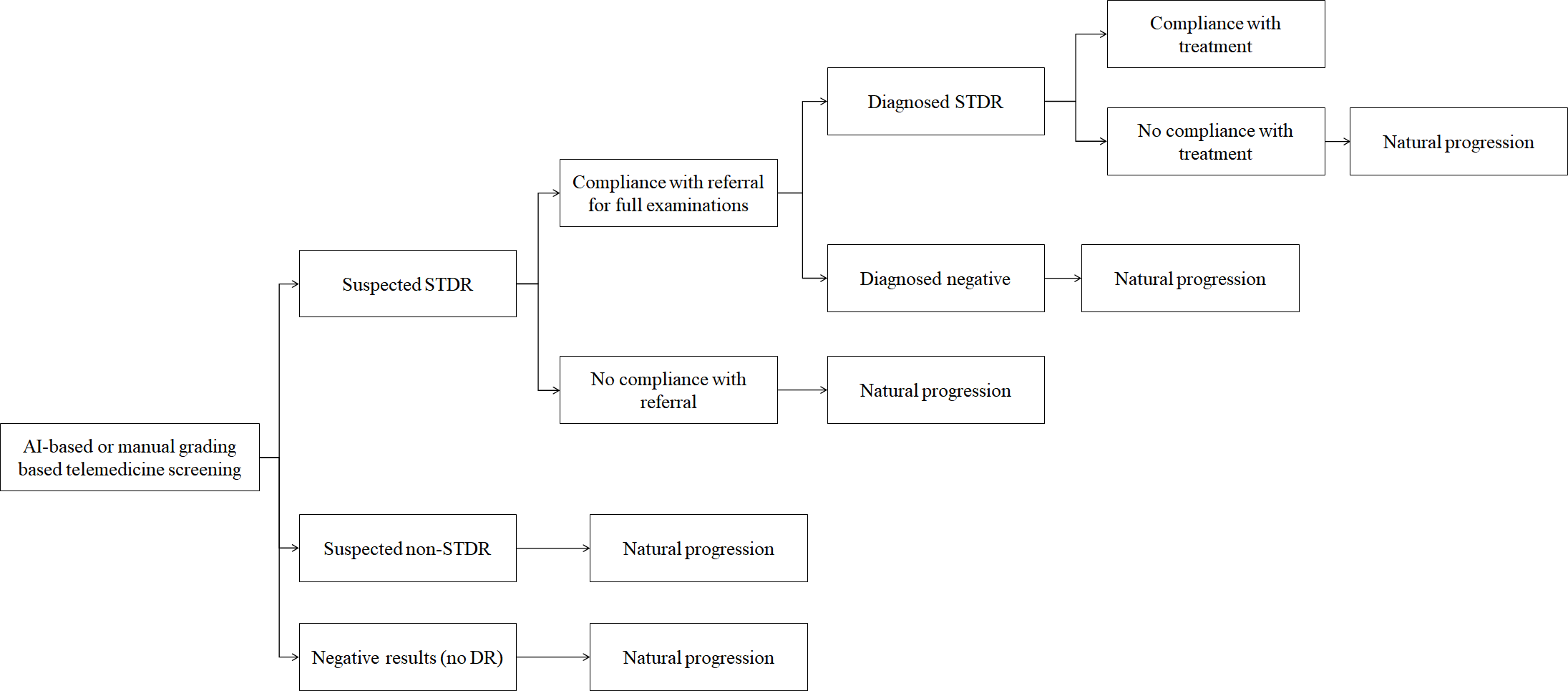

Supplement: Multimedia Appendix 5 [file publichealth_v9i1e41624_app5.docx]
