# Supplementary material for: Artificial Intelligence in Community-Based Diabetic Retinopathy Telemedicine Screening in Urban China: Cost-effectiveness and Cost-Utility Analyses With Real-world Data
Source: JMIR Public Health Surveill. 2023 Feb 23;9:e41624. doi: 10.2196/41624 (PMC9999255; doi:10.2196/41624)
Supplement: Multimedia Appendix 6 [file publichealth_v9i1e41624_app6.docx]

**Appendix 6. Cost composition of manual grading-based and AI-based telemedicine screening**

|  |  |  | **Purchase or maintenance cost** | **Rated operating years** | **Part-time staff daily salary a** | **count** | **Daily Cost (USD per day)** | **Daily Workload b (Number of screened participant)** | **Cost per screened participant (USD)** | **Manual grading-based telemedicine screening (USD)** | **AI-assisted telemedicine screening (USD)** |
| --- | --- | --- | --- | --- | --- | --- | --- | --- | --- | --- | --- |
| **Direct medical costs** | **Medical labor cost** | **On-site examination** | **/** | |  |  |  |  |  |  |  |
|  |  | *Registration (Optometrist)* |  |  | USD 43.5 (CNY 300) | 1 | 43.5 | 30 | 1.45 | Need | Need |
|  |  | *Visual acuity examination (Optometrist)* |  |  | USD 43.5 (CNY 300) | 1 | 43.5 | 30 | 1.45 | Need | Need |
|  |  | *Automatic refractor (Optometrist)* |  |  | USD 43.5 (CNY 300) | 1 | 43.5 | 30 | 1.45 | Need | Need |
|  |  | *Retinal photography (Optometrist)* |  |  | USD 50.7 (CNY 350) | 1 | 50.7 | 30 | 1.69 | Need | Need |
|  |  | **DR diagnosis** |  |  |  |  |  |  |  |  |  |
|  |  | *Diagnosis and grading (Retinal expert)* |  |  | USD 58 (CNY 400) | 2 | 116 | 100 | 1.16 | Need | Not need |
|  | **Equipment and telemedicine platform** | **On-site examination** |  |  |  |  |  |  |  |  |  |
|  |  | *Screening Registration system* | USD 14492.8 (CNY 100000) | 1 | / | 1 | 39.7 | 30 | 1.32 | Need | Need |
|  |  | Automated refractor | USD 14492.8 (CNY 100000) | 5 |  | 1 | 7.9 | 30 | 0.26 | Need | Need |
|  |  | Fundus camera | USD 43478.3 (CNY 300000) | 5 |  | 1 | 23.8 | 30 | 0.79 | Need | Need |
|  |  | Computer | USD 724.6 (CNY 5000) | 5 |  | 4 | 15.9 | 30 | 0.53 | Need | Need |
|  |  | **DR diagnosis** |  |  |  |  |  |  |  |  |  |
|  |  | AI software | USD 7246.4 (CNY 50000) | 1 |  | 1 | 19.9 | 30 | 0.66 | Not need | Need |
| **Direct non-medical costs** | **Transportation for residents** ^c^ | / | | | | | | | 0 | Need | Need |
| **Indirect costs** | **Income loss** ^c^ |  |  |  |  |  |  |  | 0 | Need | Need |
| **Societal costs** |  |  |  |  |  |  |  |  |  | 10.1 | 9.6 |

In 2020, 1 USD=6.9 CNY; extracted from State Administration of Foreign Exchange at: https://www.safe.gov.cn/safe/2020/1218/17833.html

^a^ Participation in the DR screening had no impact on full-time hospital staff salaries. Therefore, full-time hospital staff salaries did not truly reflect the labor costs of screening. However, part-time staff salaries were determined entirely based on their work content and screening workload, which could be a good reflection of labor costs. In the Shanghai DR screening program, qualified optometrists were hired for on-site examinations, while retinal experts, senior ophthalmologists specializing in retinal diseases, were hired to carry out DR diagnosis and grading in the designated diabetic retinopathy diagnosis centers.

^b^ According to our on-site observation, on average, it took 6.2, 3, 3.3 and 4.8 minutes for one participant to complete registration, visual acuity assessment, automated refraction, and retinal photography, respectively. In theory, a team with four optometrists on-site could screen nearly 100 participants daily, but under actual working conditions this is closer to 30/day. The daily workload for retinal experts under real-world conditions was about 100 participants/day.

^c^ As the community health centres in Shanghai are located in close to residential areas, we assumed that there is no transportation cost for residents to attend the screening. In addition, since the majority of the participants was older than 65 years old, so we assumed that they did not produce wage loss. The wage loss for accompanying family members were also not included in screening costs.
