# Supplementary material for: Artificial Intelligence in Community-Based Diabetic Retinopathy Telemedicine Screening in Urban China: Cost-effectiveness and Cost-Utility Analyses With Real-world Data
Source: JMIR Public Health Surveill. 2023 Feb 23;9:e41624. doi: 10.2196/41624 (PMC9999255; doi:10.2196/41624)
Supplement: Multimedia Appendix 7 [file publichealth_v9i1e41624_app7.docx]

**Appendix 7. Cost composition of full examination**

|  |  | **Price in public hospitals ^a^** | **Cost per out-patient (USD)** |
| --- | --- | --- | --- |
| **Direct medical costs** | Registration fee | USD 2.9 (CNY 20) | 2.9 |
|  | Visual acuity | USD 0.7 (CNY 5) | 0.7 |
|  | Slit lamp | USD 2.2 (CNY 15) | 2.2 |
|  | Intraocular pressure | USD 2.2 (CNY 15) | 2.2 |
|  | Fundus photography | USD 5.8 (CNY 40) | 5.8 |
|  | Fundus fluorescein angiography | USD 21.7 (CNY 150) | 21.7 |
|  | Optical coherence tomography | USD 10.1 (CNY 70 per eye) | 20.3 |
| **Direct non-medical costs** | Transportation | / | 1.2 |
| **Indirect costs ^b^** | Income loss |  | 0 |
| **Societal costs** |  |  | 57 |

In 2020, 1 USD=6.9 CNY; extracted from State Administration of Foreign Exchange at: https://www.safe.gov.cn/safe/2020/1218/17833.html

^a^ These prices are set by the Shanghai Municipal Health Commission, available at: http://wsjkw.sh.gov.cn/ylsfbz/index.html. Since public hospitals are not profit-making, money from these fees is mainly used to subsidize the cost of healthcare services. Therefore, the prices in public hospitals can be used to estimate healthcare service costs.

^b^ Indirect costs consisted of one accompanying family member’s wage loss according to time spent. However, since the majority of the participants and his/her accompanying family member was older than 65 years old, we assumed that they did not produce wage loss.
