# Supplementary material for: Artificial Intelligence in Community-Based Diabetic Retinopathy Telemedicine Screening in Urban China: Cost-effectiveness and Cost-Utility Analyses With Real-world Data
Source: JMIR Public Health Surveill. 2023 Feb 23;9:e41624. doi: 10.2196/41624 (PMC9999255; doi:10.2196/41624)
Supplement: Multimedia Appendix 8 [file publichealth_v9i1e41624_app8.docx]

**Appendix 8. Cost composition of treatment for patients with STDR**

|  | | **First year** | | | **Follow-up** | | |
| --- | --- | --- | --- | --- | --- | --- | --- |
|  |  | Severe NPDR or PDR | DME | Blindness | Severe NPDR or PDR | DME | Blindness |
| **Direct medical costs** | **Treatment ^a^** | 504.1 | 2521.7 | / | 840.6 | 840.6 | / |
|  | **Registration fee** | 2.9 | 2.9 |  | 2.9 | 2.9 |  |
|  | **Visual acuity** | 0.7 | 0.7 |  | 0.7 | 0.7 |  |
|  | **Slit lamp** | 2.2 | 2.2 |  | 2.2 | 2.2 |  |
|  | **Intraocular pressure** | 2.2 | 2.2 |  | 2.2 | 2.2 |  |
|  | **Fundus photography** | 5.8 | 5.8 |  | 5.8 | 5.8 |  |
|  | **Optical coherence tomography** | 20.3 | 20.3 |  | 20.3 | 20.3 |  |
| **Direct non-medical costs** | **Transportation** | 10.4 | 4.6 |  | 2.3 | 2.3 |  |
| **Indirect costs ^b^** | **Income loss** | 377.7 | 167.8 |  | 83.9 | 83.9 |  |
| **Societal costs** |  | 926.3 | 2728.2 | 8920^[1]^ | 960.9 | 960.9 | 3600^[1]^ |

DR= diabetic retinopathy. STDR= sight-threatening DR. NPDR= nonproliferative diabetic retinopathy. PDR= proliferative diabetic retinopathy. DME= diabetic macular edema.

^a^ In the first year, patients with DME received three times of anti-vascular endothelial growth factor treatment; photocoagulation or vitrectomy was recommended for patients with severe NPDR or PDR. In subsequent years, one time of anti-vascular endothelial growth factor treatment on average were recommended for patients with STDR. Annual outpatient review was needed for patients with STDR.

^b^ Indirect costs consisted of one accompanying family member’s wage loss according to time spent and per capita daily income in Shanghai in 2020 (https://tjj.sh.gov.cn/tjnj/nj21.htm?d1=2021tjnj/C0907.htm).

1. Li R, Yang Z, Zhang Y, Bai W, Du Y, Sun R, Tang J, Wang N, Liu H. Cost-effectiveness and cost-utility of traditional and telemedicine combined population-based age-related macular degeneration and diabetic retinopathy screening in rural and urban China. Lancet Reg Health West Pac 2022 Jun; 23:100435
