# Supplementary material for: Artificial Intelligence in Community-Based Diabetic Retinopathy Telemedicine Screening in Urban China: Cost-effectiveness and Cost-Utility Analyses With Real-world Data
Source: JMIR Public Health Surveill. 2023 Feb 23;9:e41624. doi: 10.2196/41624 (PMC9999255; doi:10.2196/41624)
Supplement: Multimedia Appendix 9 [file publichealth_v9i1e41624_app9.docx]

**Appendix 9. Markov model for DR**


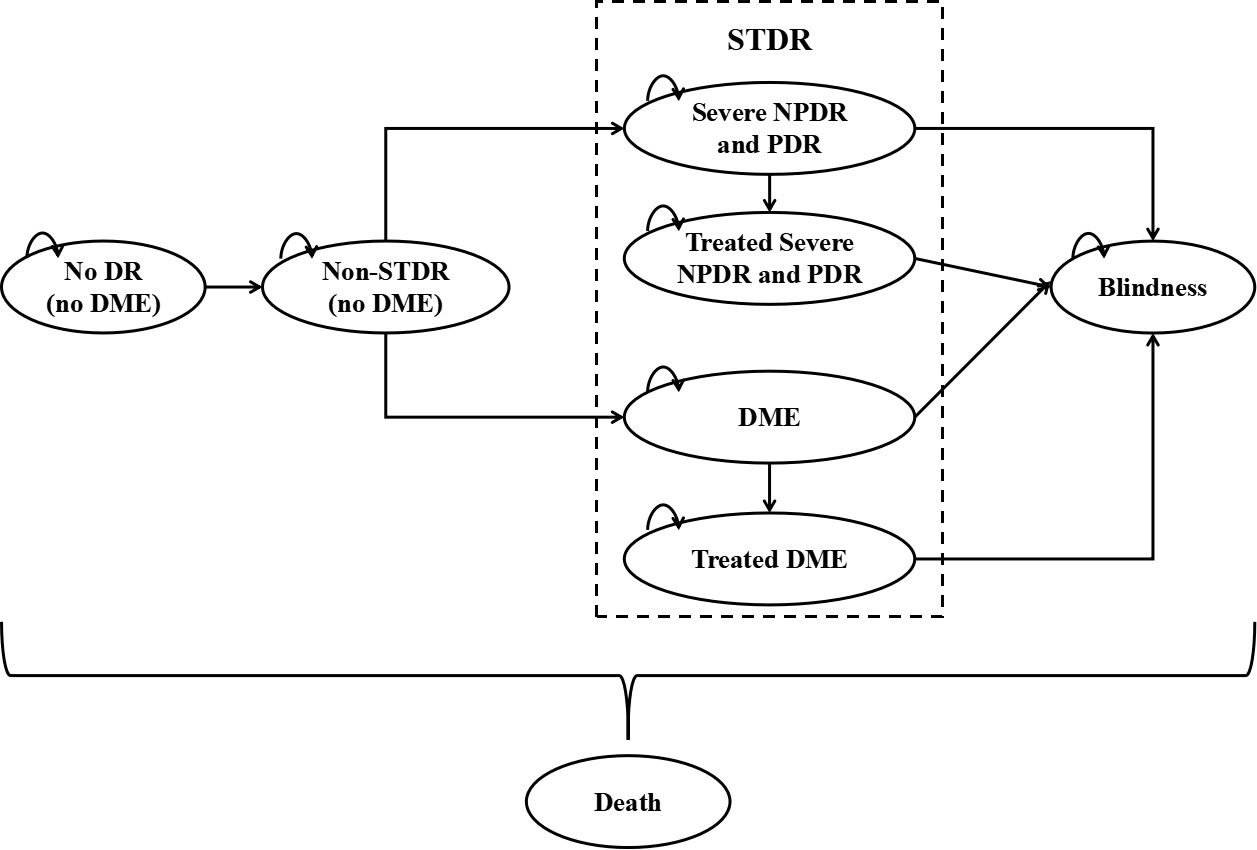


DR= diabetic retinopathy. STDR= sight-threatening DR. NPDR= nonproliferative diabetic retinopathy. PDR= proliferative diabetic retinopathy. DME= diabetic macular edema.

STDR is defined as the presence of severe NPDR, PDR, or DME, using the Eye Diseases Prevalence Research Group definition.
