# Supplementary material for: Artificial Intelligence in Community-Based Diabetic Retinopathy Telemedicine Screening in Urban China: Cost-effectiveness and Cost-Utility Analyses With Real-world Data
Source: JMIR Public Health Surveill. 2023 Feb 23;9:e41624. doi: 10.2196/41624 (PMC9999255; doi:10.2196/41624)
Supplement: Multimedia Appendix 10 [file publichealth_v9i1e41624_app10.docx]

**Appendix 10. Accuracy of AI-assisted model using in Shanghai**

| ***Classification*** | **Real disease states** | **Diagnosis results** | **Probability** |
| --- | --- | --- | --- |
| Binary | No STDR^a^ | No STDR^a^ | 97.96% |
|  |  | STDR | 2.04% |
|  | STDR | No STDR^a^ | 19.53% |
|  |  | STDR | 80.47% |
| Multiple | No DR | No DR | 74.45% |
|  |  | Non-STDR | 25.55% |
|  |  | STDR | 0% |
|  | Non-STDR | No DR | 2.61% |
|  |  | Non-STDR | 93.36% |
|  |  | STDR | 4.03% |
|  | STDR | No DR | 0.39% |
|  |  | Non-STDR | 19.14% |
|  |  | STDR | 80.47% |

DR= diabetic retinopathy. STDR= sight-threatening DR. NPDR= nonproliferative diabetic retinopathy. PDR= proliferative diabetic retinopathy. DME= diabetic macular edema.

^a^ including no DR and non-STDR.
