# Supplementary material for: Artificial Intelligence in Community-Based Diabetic Retinopathy Telemedicine Screening in Urban China: Cost-effectiveness and Cost-Utility Analyses With Real-world Data
Source: JMIR Public Health Surveill. 2023 Feb 23;9:e41624. doi: 10.2196/41624 (PMC9999255; doi:10.2196/41624)
Supplement: Multimedia Appendix 12 [file publichealth_v9i1e41624_app12.docx]

**Appendix 12. Deterministic sensitivity analysis result of change of compliance with referral after the adoption of AI (Multiplier)**

|  | **Multiplier** | **Strategy** | **Cost** | **Effectiveness** | **Incremental cost** | **Incremental effectiveness** | **ICER or ICUR value** | **Dominated** |
| --- | --- | --- | --- | --- | --- | --- | --- | --- |
| ICER | 0.75 | AI | 3218.57 | 9.76 | N/A | N/A | N/A |  |
|  | 0.75 | Manual grading | 3265.37 | 9.83 | 46.80 | 0.07 | 635.63 |  |
|  | 0.775 | AI | 3214.62 | 9.76 | N/A | N/A | N/A |  |
|  | 0.775 | Manual grading | 3265.37 | 9.83 | 50.75 | 0.07 | 734.63 |  |
|  | 0.8 | AI | 3210.75 | 9.77 | N/A | N/A | N/A |  |
|  | 0.8 | Manual grading | 3265.37 | 9.83 | 54.62 | 0.06 | 844.95 |  |
|  | 0.825 | AI | 3206.95 | 9.77 | N/A | N/A | N/A |  |
|  | 0.825 | Manual grading | 3265.37 | 9.83 | 58.41 | 0.06 | 968.60 |  |
|  | 0.85 | AI | 3203.24 | 9.78 | N/A | N/A | N/A |  |
|  | 0.85 | Manual grading | 3265.37 | 9.83 | 62.13 | 0.06 | 1108.13 |  |
|  | 0.875 | AI | 3199.60 | 9.78 | N/A | N/A | N/A |  |
|  | 0.875 | Manual grading | 3265.37 | 9.83 | 65.77 | 0.05 | 1266.79 |  |
|  | 0.9 | AI | 3196.04 | 9.79 | N/A | N/A | N/A |  |
|  | 0.9 | Manual grading | 3265.37 | 9.83 | 69.33 | 0.05 | 1448.74 |  |
|  | 0.925 | AI | 3192.54 | 9.79 | N/A | N/A | N/A |  |
|  | 0.925 | Manual grading | 3265.37 | 9.83 | 72.82 | 0.04 | 1659.48 |  |
|  | 0.95 | AI | 3189.12 | 9.79 | N/A | N/A | N/A |  |
|  | 0.95 | Manual grading | 3265.37 | 9.83 | 76.25 | 0.04 | 1906.39 |  |
|  | 0.975 | AI | 3185.76 | 9.80 | N/A | N/A | N/A |  |
|  | 0.975 | Manual grading | 3265.37 | 9.83 | 79.61 | 0.04 | 2199.59 |  |
|  | 1 | AI | 3182.47 | 9.80 | N/A | N/A | N/A |  |
|  | 1 | Manual grading | 3265.37 | 9.83 | 82.90 | 0.03 | 2553.39 |  |
|  | 1.025 | AI | 3179.24 | 9.80 | N/A | N/A | N/A |  |
|  | 1.025 | Manual grading | 3265.37 | 9.83 | 86.13 | 0.03 | 2988.65 |  |
|  | 1.05 | AI | 3176.07 | 9.81 | N/A | N/A | N/A |  |
|  | 1.05 | Manual grading | 3265.37 | 9.83 | 89.29 | 0.03 | 3537.07 |  |
|  | 1.075 | AI | 3172.97 | 9.81 | N/A | N/A | N/A |  |
|  | 1.075 | Manual grading | 3265.37 | 9.83 | 92.40 | 0.02 | 4249.24 |  |
|  | 1.1 | AI | 3169.92 | 9.81 | N/A | N/A | N/A |  |
|  | 1.1 | Manual grading | 3265.37 | 9.83 | 95.45 | 0.02 | 5211.22 |  |
|  | 1.125 | AI | 3166.93 | 9.82 | N/A | N/A | N/A |  |
|  | 1.125 | Manual grading | 3265.37 | 9.83 | 98.44 | 0.01 | 6582.15 |  |
|  | 1.15 | AI | 3163.99 | 9.82 | N/A | N/A | N/A |  |
|  | 1.15 | Manual grading | 3265.37 | 9.83 | 101.38 | 0.01 | 8692.82 |  |
|  | 1.175 | AI | 3161.11 | 9.82 | N/A | N/A | N/A |  |
|  | 1.175 | Manual grading | 3265.37 | 9.83 | 104.26 | 0.01 | 12362.48 |  |
|  | 1.2 | AI | 3158.28 | 9.83 | N/A | N/A | N/A |  |
|  | 1.2 | Manual grading | 3265.37 | 9.83 | 107.09 | 0.01 | 20328.23 |  |
|  | 1.225 | AI | 3155.50 | 9.83 | N/A | N/A | N/A |  |
|  | 1.225 | Manual grading | 3265.37 | 9.83 | 109.87 | 0.00 | 50774.83 |  |
|  | 1.25 | AI | 3152.77 | 9.83 | N/A | N/A | N/A |  |
|  | 1.25 | Manual grading | 3265.37 | 9.83 | 112.60 | 0.00 | -127850.29 | Dominated |
| ICUR | 0.75 | AI | 3218.57 | 6.74 | N/A | N/A | N/A |  |
|  | 0.75 | Manual grading | 3265.37 | 6.75 | 46.80 | 0.01 | 3799.52 |  |
|  | 0.775 | AI | 3214.62 | 6.74 | N/A | N/A | N/A |  |
|  | 0.775 | Manual grading | 3265.37 | 6.75 | 50.75 | 0.01 | 4389.86 |  |
|  | 0.8 | AI | 3210.75 | 6.74 | N/A | N/A | N/A |  |
|  | 0.8 | Manual grading | 3265.37 | 6.75 | 54.62 | 0.01 | 5047.42 |  |
|  | 0.825 | AI | 3206.95 | 6.74 | N/A | N/A | N/A |  |
|  | 0.825 | Manual grading | 3265.37 | 6.75 | 58.41 | 0.01 | 5784.24 |  |
|  | 0.85 | AI | 3203.24 | 6.74 | N/A | N/A | N/A |  |
|  | 0.85 | Manual grading | 3265.37 | 6.75 | 62.13 | 0.01 | 6615.44 |  |
|  | 0.875 | AI | 3199.60 | 6.74 | N/A | N/A | N/A |  |
|  | 0.875 | Manual grading | 3265.37 | 6.75 | 65.77 | 0.01 | 7560.28 |  |
|  | 0.9 | AI | 3196.04 | 6.75 | N/A | N/A | N/A |  |
|  | 0.9 | Manual grading | 3265.37 | 6.75 | 69.33 | 0.01 | 8643.58 |  |
|  | 0.925 | AI | 3192.54 | 6.75 | N/A | N/A | N/A |  |
|  | 0.925 | Manual grading | 3265.37 | 6.75 | 72.82 | 0.01 | 9898.04 |  |
|  | 0.95 | AI | 3189.12 | 6.75 | N/A | N/A | N/A |  |
|  | 0.95 | Manual grading | 3265.37 | 6.75 | 76.25 | 0.01 | 11367.48 |  |
|  | 0.975 | AI | 3185.76 | 6.75 | N/A | N/A | N/A |  |
|  | 0.975 | Manual grading | 3265.37 | 6.75 | 79.61 | 0.01 | 13112.12 |  |
|  | 1 | AI | 3182.47 | 6.75 | N/A | N/A | N/A |  |
|  | 1 | Manual grading | 3265.37 | 6.75 | 82.90 | 0.01 | 15216.96 |  |
|  | 1.025 | AI | 3179.24 | 6.75 | N/A | N/A | N/A |  |
|  | 1.025 | Manual grading | 3265.37 | 6.75 | 86.13 | 0.00 | 17806.07 |  |
|  | 1.05 | AI | 3176.07 | 6.75 | N/A | N/A | N/A |  |
|  | 1.05 | Manual grading | 3265.37 | 6.75 | 89.29 | 0.00 | 21067.90 |  |
|  | 1.075 | AI | 3172.97 | 6.75 | N/A | N/A | N/A |  |
|  | 1.075 | Manual grading | 3265.37 | 6.75 | 92.40 | 0.00 | 25303.25 |  |
|  | 1.1 | AI | 3169.92 | 6.75 | N/A | N/A | N/A |  |
|  | 1.1 | Manual grading | 3265.37 | 6.75 | 95.45 | 0.00 | 31023.66 |  |
|  | 1.125 | AI | 3166.93 | 6.75 | N/A | N/A | N/A |  |
|  | 1.125 | Manual grading | 3265.37 | 6.75 | 98.44 | 0.00 | 39175.28 |  |
|  | 1.15 | AI | 3163.99 | 6.75 | N/A | N/A | N/A |  |
|  | 1.15 | Manual grading | 3265.37 | 6.75 | 101.38 | 0.00 | 51724.65 |  |
|  | 1.175 | AI | 3161.11 | 6.75 | N/A | N/A | N/A |  |
|  | 1.175 | Manual grading | 3265.37 | 6.75 | 104.26 | 0.00 | 73542.35 |  |
|  | 1.2 | AI | 3158.28 | 6.75 | N/A | N/A | N/A |  |
|  | 1.2 | Manual grading | 3265.37 | 6.75 | 107.09 | 0.00 | 120900.52 |  |
|  | 1.225 | AI | 3155.50 | 6.75 | N/A | N/A | N/A |  |
|  | 1.225 | Manual grading | 3265.37 | 6.75 | 109.87 | 0.00 | 301908.66 |  |
|  | 1.25 | AI | 3152.77 | 6.75 | N/A | N/A | N/A |  |
|  | 1.25 | Manual grading | 3265.37 | 6.75 | 112.60 | 0.00 | -760027.34 | Dominated |

AI=artificial intelligence. ICER= incremental cost-effectiveness ratio. ICUR= incremental cost-utility ratio.
