# Supplementary material for: Artificial Intelligence in Community-Based Diabetic Retinopathy Telemedicine Screening in Urban China: Cost-effectiveness and Cost-Utility Analyses With Real-world Data
Source: JMIR Public Health Surveill. 2023 Feb 23;9:e41624. doi: 10.2196/41624 (PMC9999255; doi:10.2196/41624)
Supplement: Multimedia Appendix 13 [file publichealth_v9i1e41624_app13.docx]

**Appendix 13. Sensitivity analysis results for ICER ^a^**

| **Parameter** | **Lowest ICER** | **Highest ICER** | **Range of ICER change** |
| --- | --- | --- | --- |
| Prevalence of non-STDR at the baseline | 2673.9 | 2709.5 | 35.6 |
| Prevalence of severe NPDR and PDR at the baseline | 2678.3 | 2705.4 | 27.1 |
| Prevalence of DME at the baseline | 2665.3 | 2717.2 | 52.0 |
| Prevalence of blindness at the baseline | 2691.4 | 2691.4 | 0.0 |
| Transitional probability: no DR to non-STDR | 2675.9 | 2707.8 | 31.9 |
| Transitional probability: non-STDR to severe NPDR and PDR | 2507.9 | 2857.9 | 350.0 |
| Transitional probability: non-STDR to DME | 2535.6 | 2841.9 | 306.3 |
| Transitional probability: untreated severe NPDR and PDR to blindness | 2419.2 | 2985.3 | 566.1 |
| Transitional probability: untreated DME to blindness | 2377.6 | 3058.3 | 680.7 |
| Transitional probability: treated severe NPDR and PDR to blindness | 2566.1 | 2821.5 | 255.5 |
| Transitional probability: treated DME to blindness | 2484.8 | 2911.8 | 427.0 |
| Compliance with referral | 2641.3 | 2744.0 | 102.8 |
| Compliance with treatment | 2642.2 | 2746.9 | 104.6 |
| Utility value of no DR | 2691.4 | 2691.4 | 0.0 |
| Utility value of non-STDR | 2691.4 | 2691.4 | 0.0 |
| Utility value of STDR | 2691.4 | 2691.4 | 0.0 |
| Utility value of blindness | 2691.4 | 2691.4 | 0.0 |
| Accuracy of AI-assisted model: no DR called STDR | 2650.9 | 2691.4 | 40.5 |
| Accuracy of AI-assisted model: non-STDR called STDR | 2691.4 | 2712.0 | 20.6 |
| Accuracy of AI-assisted model: STDR called STDR | 2691.4 | 2892.4 | 201.1 |
| Accuracy of manual grading: no DR called STDR | 2691.4 | 2731.9 | 40.5 |
| Accuracy of manual grading: non-STDR called STDR | 2683.1 | 2699.6 | 16.5 |
| Accuracy of manual grading: STDR called STDR | 2654.9 | 2822.0 | 167.1 |
| Costs of on-stie screening in manual grading based telemedicine screening | 1546.7 | 3836.0 | 2289.3 |
| Costs of on-stie screening in AI-based telemedicine screening | 1588.8 | 3794.0 | 2205.1 |
| Costs off full ophthalmologic examination | 2687.0 | 2695.8 | 8.8 |
| Treatment costs for the first year: Severe NPDR and PDR | 2480.7 | 2902.1 | 421.4 |
| Treatment costs for the first year: DME | 2009.6 | 3428.4 | 1418.9 |
| Treatment costs for the first year: Blindness | 2057.1 | 3325.3 | 1268.2 |
| Treatment costs for the follow-up: Severe NPDR and PDR | 1850.4 | 3532.3 | 1681.9 |
| Treatment costs for the follow-up: DME | 1741.1 | 3641.6 | 1900.5 |
| Treatment costs for the follow-up: Blindness | 1927.3 | 3455.8 | 1528.4 |

DR= diabetic retinopathy. STDR= sight-threatening DR. NPDR= nonproliferative diabetic retinopathy. PDR= proliferative diabetic retinopathy.

DME= diabetic macular edema. ICER= incremental cost-effectiveness ratio. ICUR= incremental cost-utility ratio.

^a^ Since negative values of ICER or ICUR might occur due to the change of compliance with referral after the adoption of AI (Multiplier), detailed results have been displayed in Appendix 13 separately. Therefore, in this appendix, the results of the change of compliance with referral after the adoption of AI (Multiplier) have no longer displayed.
